# Current and future aspects of IBD research and treatment: The 2022 perspective

**DOI:** 10.3389/fgstr.2022.914371

**Published:** 2022-08-11

**Authors:** Eduard F. Stange

**Affiliations:** Department of Internal Medicine I, Medical University Hospital, Tübingen, Germany

**Keywords:** Crohn´s disease, ulcerative colitis, pathogenesis, defensins, treatment

## Abstract

Inflammatory bowel diseases (IBD) have seen major progress in current concepts and treatment regimes. Based on the theory of an inadequate “overshoot” of the mucosal immune response to the intestinal microbiome, therapies have been developed to interfere with the key mediators of inflammation from cytokines, including TNF and IL12/23, to integrins such as α4ß7 and intracellular cytokine signal transducers such as janus kinases. Recently, sphingosine-1-receptor agonists were marketed to suppress mucosal inflammation by sequestering lymphocytes in peripheral lymph nodes. However, the aim of these regimes targeting immunity to induce a long-term deep remission, including mucosal healing, is missed in most patients. Contrasting these anti-inflammatory mechanisms of action, the pathogenic focus has finally shifted to the mucosal antibacterial barrier in both Crohn´s disease and ulcerative colitis. Translating this novel concept requires a completely different approach but, in the end, may come closer to a cure of these devastating diseases, in which an incomplete immune modulation fails to achieve the key endpoints: halting disease activity and progression. This review aims to give an overview of past, current, and future concepts in IBD, focusing on both pathogenesis and consequent therapy. A cure is in sight only if both reflect the actual key mechanisms of slow bacterial entry into the mucosa and are harmonized and in line.

## Introduction

The treatment of gastric and duodenal ulcer disease as well as former non-A/non-B hepatitis has been revolutionized by identification and targeting of the culprit: helicobacter pylori and hepatitis C virus. In both instances, it is justified to use the term “cure.” In contrast, therapeutic strategies in inflammatory bowel diseases (IBD) are unsatisfactory, hitting a low ceiling of treatment success (1, 2), and even therapeutic targets are heavily debated (3). Progress is significant but limited: response rates of so-called biologicals are still suboptimal, and the hype (1) as well as the enormous cost of biologicals has not translated into satisfying and predictable long-term benefit to patients, even in responders. Due to antibody formation and other unknown mechanisms, loss of response is a major problem, leading to dose hikes or switches of the biological. Similarly, the benefit of JAK-inhibitors is very limited and again helpful in only a minority of patients (4).

Independent of treatment specifics, only a minority of patients indeed achieve long-term remission, and the treatment outcome cannot be predicted from clinical, laboratory, genetic, or microbial parameters at the start. Therefore, it is difficult to choose the “right” medication for any individual patient, and the hapless doctor is left with a blind effort of trial and error. Why is this so, what about the “pathogenetic culprit,” and is there a positive prospect for a “cure”?

At times, it is helpful (and necessary) to lean back and screen the horizon, on which the concept of an inadequate “overshoot” of the (adaptive) immune response originated and why this concept has led the field astray. So what are the best alternatives to move forward? It seems likely that more of the same, such as new kinds of immune modulation (5), will not provide the kind of remedy for which the IBD field and, above all, patients are looking. Using fecal microbiome transfer, the alternative approach to modulating the microbiome, as a potential aggressor has also attained only limited success in about a quarter of patients (6). Rather, the defective mucosal and mucus antibacterial barrier in both Crohn´s disease (CD) and ulcerative colitis (UC) should enter the focus, not just of basic pathogenetic research, but also as a treatment target (7).

The following, admittedly eclectic, account only roughly follows chronological order because pathogenetic and consequent therapeutic reasoning in IBD has rarely been chronological and sometimes not even logical. Also, novel therapeutic developments in the past were not necessarily based on etiological insight, and until recently, there has been an unfortunate dissociation of both.

## The past

### Standard therapy: key trials and other evidence

A brief look at IBD history (8, 9) tells us that the first serious trial was a by-product of investigations on the treatment of rheumatoid arthritis (RA) using a novel synthetic drug called salazopyrine (salicylazosulfapyridine, SASP) (10). The drug was designed by Nana Svartz at the Karolinska Institute supposedly through an “act of female intuition devoid of any logic” (quote from Sidney Truelove). In contrast to this comment (the term “male chauvinist” was not yet in use), she had indeed successfully aimed at the combination of an anti-inflammatory (5-aminosalicylic acid) and antibacterial (sulfapyridine). At the time, she believed that RA was caused by bacteria also present in milk, and although this turned out to be wrong, the drug worked in RA and also in UC: in UC, 75 of 124 patients were free from subjective symptoms, and 41 had improved; only eight failures were reported (10). Many years later, it turned out that SASP was only a pro-drug and the active moiety was 5-aminosalicylic acid (5-ASA). In UC, it became, and still is, the drug of choice for induction to avoid steroids in mild-to-moderate disease and also for maintaining remission (11). The drug SASP is also shown to have limited benefit in CD, particularly in the colon, and the effect on CD of 5-ASA is still a matter of debate although the higher doses may well be effective (11).

Without giving a pathogenetic rationale, Sidney Truelove, together with his colleague Lesley John Witts from Oxford in the United Kingdom, started a controlled, blinded trial on cortisone in UC in 1952, and the final results were published in 1955 (12). A remarkable 213 patients were treated, and the approximately half that were treated with variable doses of cortisone fared significantly better than the controls: nearly 70% achieved remission or were improved compared with about 40%, respectively. The effect was superior in a first attack vs. relapse, and most importantly, the excessive mortality in those days was down from about 24% to 8%. This study proved to be revolutionary in two aspects: introducing solid evidence for corticosteroids in UC, later confirmed by even larger trials in CD, and also a study format that proved to stand the test of time. Now, we call it evidence-based medicine.

Much smaller case series initially suggested the use of the antimetabolites in UC. R.H.D. Bean from Australia had treated only seven patients as published in the British Medical Journal (13) with mercaptopurine, thioguanine (much later to be revived), and also busulphan. The positive findings were later confirmed by controlled trials in UC and also in CD. Other immunosuppressants introduced into the IBD field were methotrexate in CD but not UC as well as cyclosporine and tacrolimus in UC but not in CD. With the exception of calcineurin inhibitors, which are directed specifically at the IL-2 pathway, all conventional immunomodulatory approaches, therefore, were broad and unspecific. At any rate, there was strong support from the treatment benefit of immunosuppressants to suggest an immunological disease, and it seems that, in those days, the therapeutic success of steroids and antimetabolites had an impact on the pathogenetic concept, maybe more than vice versa (14). An overview of these traditional therapies is given in **Table 1**.

**Table 1:** Key points 1.

Traditional standard therapies Salizylazosulfapyridine/5-Aminosalcylic acid Corticosteroids Azathioprine/mercaptopurine Methotrexate Calcineurin inhibitors
Old pathogenetic concepts Specific infections Vasculitis Defective mucus layer Autoimmunity Hyper-response of adaptive immunity

### Old pathogenetic concept

Initially, UC was clearly differentiated from granulomatous “cicatrizing” enteritis, which today we would probably call CD of the colon (8, 9). Gross appearance and microscopic findings of lymphocyte, plasma cell, eosinophil, and macrophage infiltration in the mucosa were described in detail, but the chapter on etiology remains speculative: specific infection by *diplococcus* or *Bacterium necrophorum*, psychogenic factors, colon spasm, or mucosal entry of proteolytic enzymes were considered potential causative factors. Others believed that UC was caused by a reduction in the mucus layer above the enterocytes.

**Figure 1:**
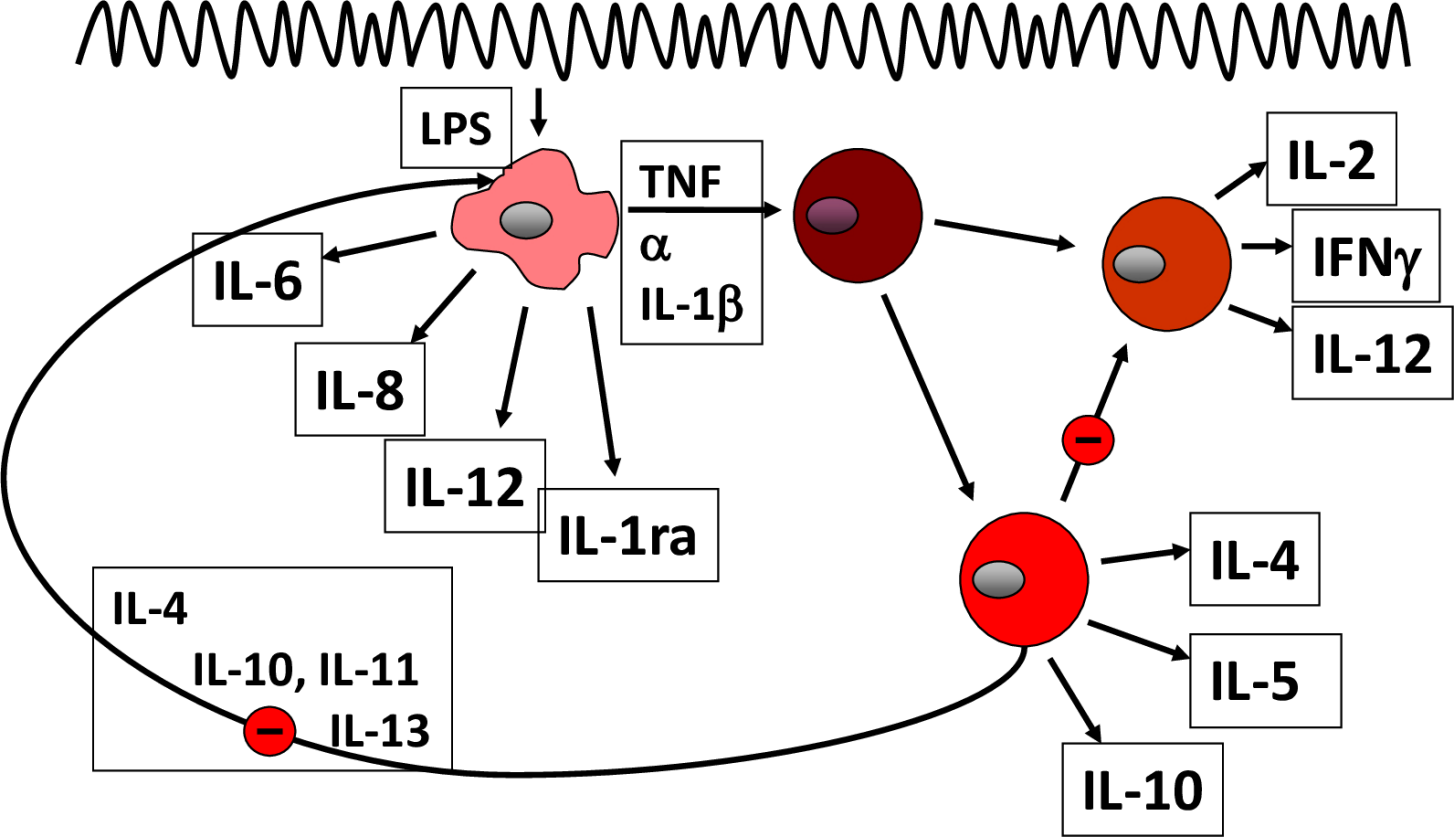
Pathogenetic concept based on cytokines.

Rather than looking at this barrier in more detail, the field entered an era of intensive lymphocyte research, and adaptive immunity and cytokines were the focus (**Table 1** and **Figure 1**). CD4+ and CD8+ lymphocytes were identified and found in the mucosa, and subsequently, the TH1 and TH2 concept was developed and applied to CD and UC, respectively. Entering the terms “autoimmunity and UC” in a Pubmed search results in 2051 results, the first mention dating back to 1959 (8) and increasing over the years until today. The basic principle of the “immunological hypothesis” maintains that IBD represents an abnormal immune response to a normal stimulus in a genetically susceptible host. This is compatible with the “overshoot” idea and would indeed move the lymphocyte into the limelight as the “culprit” (15).

In essence, the normal mucosal environment is anti-inflammatory, dominated by tolerance (15). Dendritic cells capture antigens from the lumen and present them to naïve T lymphocytes, but in the healthy state, through generation of retinoic acid and TGF-ß, the generation of T reg(ulatory) cells is promoted that downregulates the host immune response. Similarly, normal mucosal macrophages produce large amounts of anti-inflammatory IL-10; otherwise, everybody would develop IBD rather than a healthy tolerance. In IBD (**Figure 1**), activated macrophages produce tumor necrosis factor (TNF), interleukin-6, -12, and -23, promoting inflammation (14). CD4+ T cells proliferate and differentiate into effector T cell subsets, including Th1 and Th17 cells that upregulate chemokine receptors and integrins. In this regard, the integrin α4ß7 is relatively specific for the gut, and together with its partner mucosal addressin cell adhesion molecule 1 (MaDCAM1), mediates lymphocyte entry into the mucosa. Following activation, they secrete several proinflammatory cytokines, such as, IL-12, IL-17 A, IL-22 and interferon-γ, which interact with innate lymphoid cells (ILC) as well as IgG-secreting plasma cells. IL-12 and IL-23 also drive inflammation and, in particular, the differentiation of CD4+ T-cell subsets. Cytokines dock to their respective receptors and usually signal through the JAK/Stat pathways to elicit an inflammatory response of the target cell (15). The lymphocyte egress from the lymph nodes, as a matter of fact, is also regulated through appropriate receptors (sphingosine-1-phosphate receptors, S1PR).

Arguments for adaptive immunity were, for example, the benefit of bone marrow transplantation in some patients, but long-term experience reveals that relapse after varying intervals is the rule, and controlled trials were unconvincing (16). This would be quite compatible with only transient elimination of primed-to cells recovering after a time of continued stimulation. Most importantly, but very simply, the segmental localization of CD is not compatible with the adaptive immunity concept, similar to the distal start of UC migrating proximally. Both rather suggest an impact of local factors: why should “auto-aggressive” lymphocytes exclusively migrate into certain stretches of intestine but not others? Apparently, most localizations of CD (ileal, ileocecal, or colonic) are stable over time. And why should a fecal stream diversion prevent relapse in operated CD (17) and intestinal contents provoke lesions if autoimmunity was directed toward the tissue?

## The present

The quest for improved medications, of course, continued due to the limitations of standard treatments, which, at the time (around 2006), predominantly included aminosalicylates, steroids, and immunosuppressants such as thiopurines (18, 19). Sulfasalazin-induced intolerance in many patients was largely bypassed by a switch to the active moiety 5-aminosalicylic acid. However, 5-ASA was only effective in mild-to-moderate cases of acute UC, in maintaining remission in UC, and only marginally in CD. Although quite effective in the acute phase with remission rates up 85% in both IBDs, corticosteroids exhibit well-known serious side effects and, above all, fail to maintain remission. The less toxic budesonide is also less active and, again, fails to maintain remission. Both thiopurines, azathioprine and 6-mercaptopurine, suffer from potentially severe adverse events, such as bone marrow toxicity, pancreatitis, and hepatitis and, also, are effective late and only in some of the patients in maintaining remission. The same limitation applies to methotrexate, effective only marginally in CD but not in UC. On the other hand, calcineurin inhibitors are quite effective in controlling acute severe colitis but do not maintain remission and are ineffective in CD.

Despite the obvious limitations of an adaptive immunity-based concept, real progress resulted from ever more detailed molecular investigations of the cytokine mediators and of the lymphocyte integrins/endothelial adhesion molecules, mediating transendothelial entry of inflammatory cells into the mucosa. Following failure of the IL-10 trials as an attempt to directly apply an “anti-inflammatory” cytokine, the first successful target was TNFα, following development of infliximab, a neutralizing monoclonal (hybrid) antibody (20). Later, this was complemented by other anti-TNFs, such as adalimumab and golimumab. This approach was successful not through straight neutralization by binding of free, soluble TNF, as expected, but mostly through induction of apoptosis of inflammatory cells carrying TNF in the membrane-bound form. This became obvious after a specific TNF antibody (etanercept) failed to induce apoptosis (21) and remission in CD but was clinically effective in RA.

Later, an antibody binding the common p40 subunit of IL-12 and IL-23 (ustekinumab) was also clinically effective in both CD and UC as well as an antibody (vedolizumab) directed against α4ß7-integrin, blocking lymphocyte entry into the gut (1). Other antibodies, including secukinumab blocking IL-17A, not only failed in CD, but actually worsened the disease course (22). More recently, on the oral side, new medications have been developed interfering with the JAK-pathway in CD and UC (4). The final entry into the field was ozanimod, a S1PR-blocker limiting lymphocyte exit from lymph nodes (23).

Thus, research performed in this era may not have revealed the “cause” of IBD, but contributed significantly to current treatment strategies. The introduction of biologicals and JAK-inhibitors changed the therapeutic scene completely but was also accompanied by a marketing hype (1). Approval through medical agencies in both in Europe and the United States, was granted to every drug that was statistically superior to placebo, independent of the (often marginal) effect size. Therefore, the pros and cons should be weighed critically based on the key trials and other evidence, preferably industry independent.

### Current therapy: key trials and other evidence

**Figure 2:**
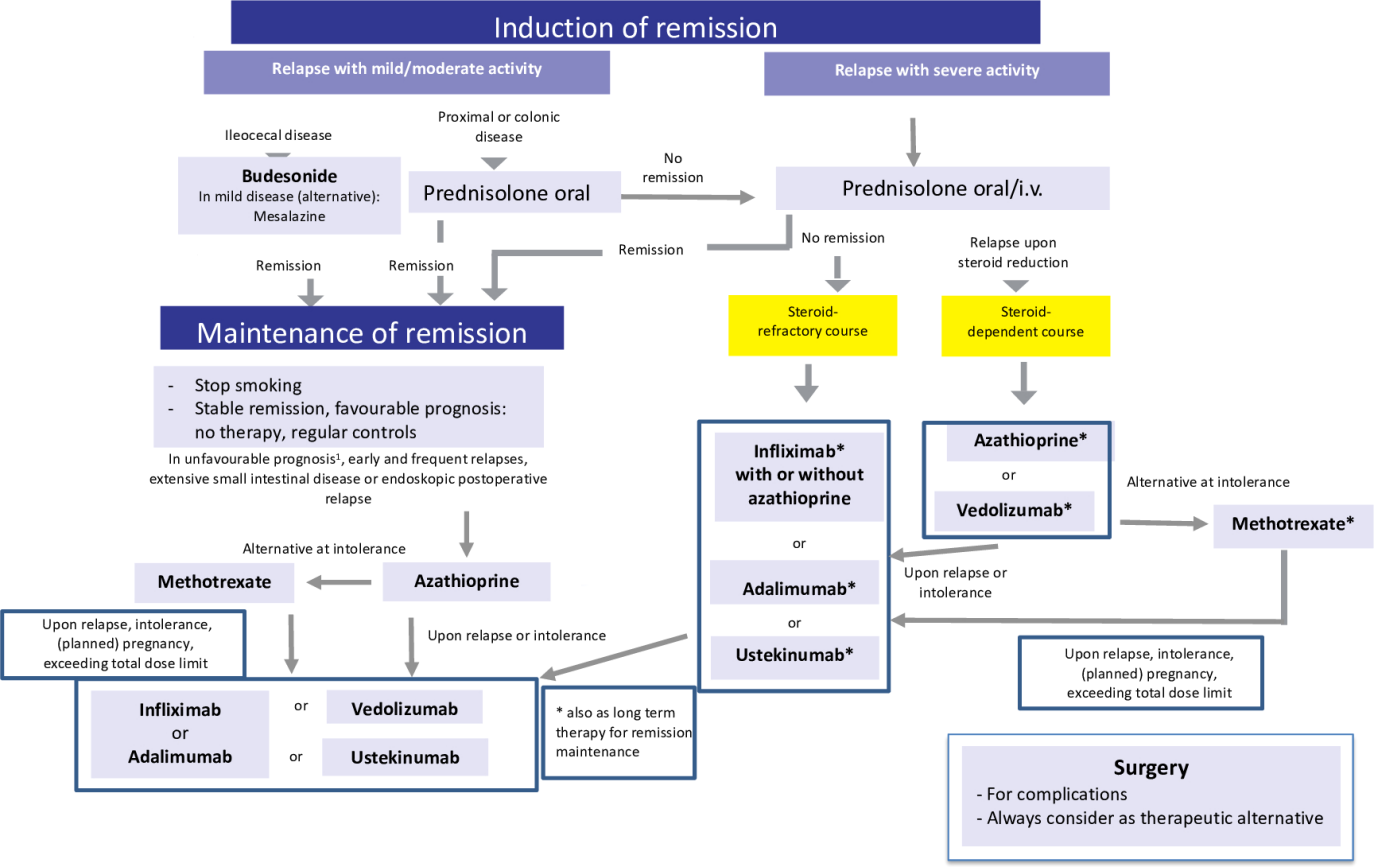
Current therapeutic algorithm of CD. Adapted from Herrlinger and Stange (1).

**Figure 3:**
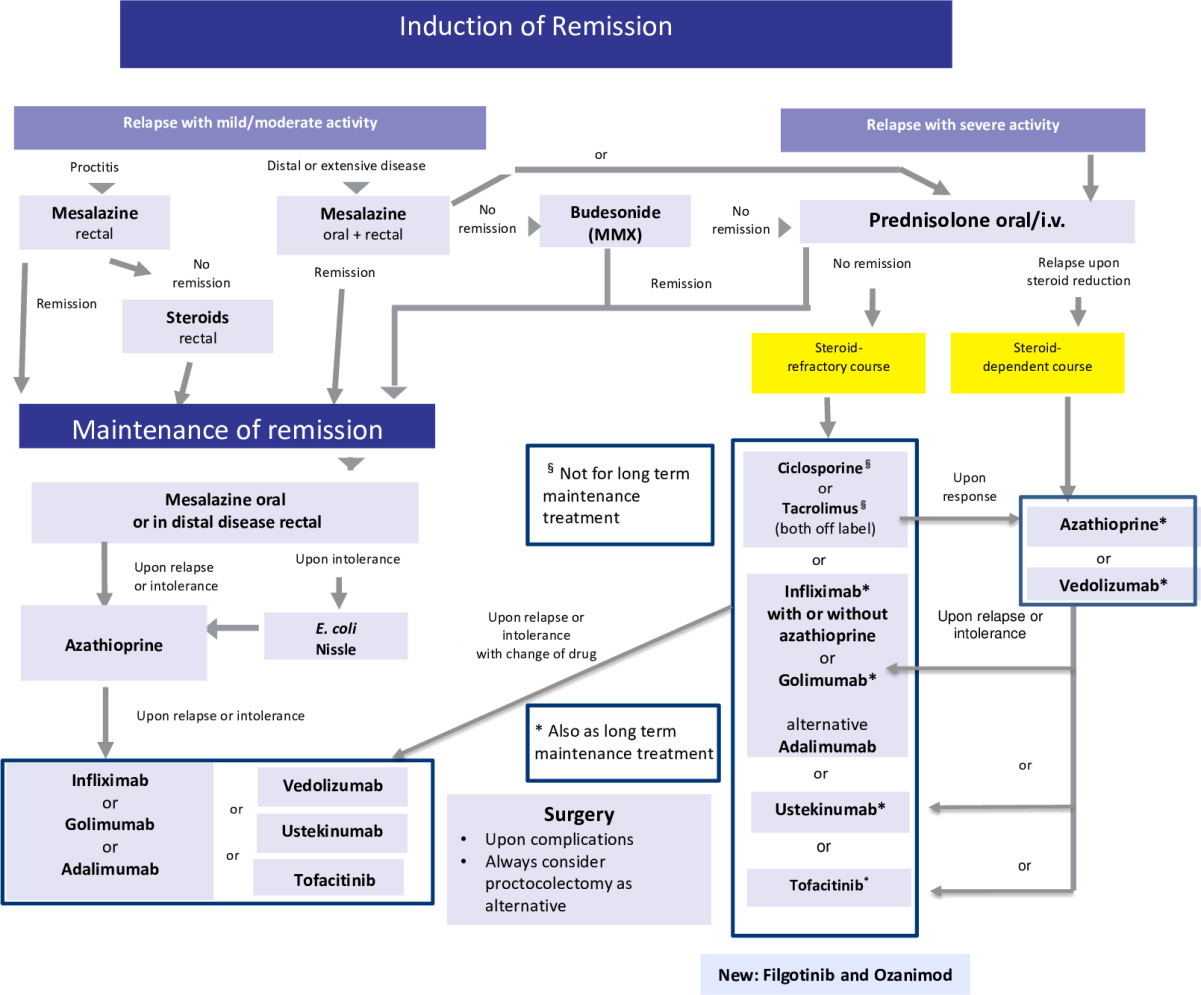
Current therapeutic algorithm of UC. Adapted from Herrlinger and Stange (1).

For an overview of current therapies, see **Table 2** and the algorithms in **Figures 2**, **3**. With the exception of golimumab (approved only for UC), all monoclonal antibodies are shown to be superior to placebo in phase 3 controlled trials in both CD and UC (11, 24). In UC, infliximab was not only the first biological to show efficacy, but also the most effective with close to 40% of UC patients achieving remission following induction. In comparison, the remission rates when using the other antibodies was much lower with <20% and the therapeutic gain was in the range of around 10% (equivalent to a number needed to treat, NNT) of 10: on average, 10 patients have to be treated to achieve one additional remission compared with placebo (1). Tofacitinib efficacy in the Octave 1 and 2 trials was in the same low range (1), and this also applies to ozanimod with a mere 18% (NNT 8) achieving remission (23).

**Table 2:** Key points 2

Current therapies Infliximab, adalimumab, golimumab (anti-TNF antibodies) Vedolizumab (anti-integrin) Ustekinumab (anti-IL12/23) Tofacitinib, filgotinib (JAK-Inhibitors) Ozanimod (sphingosine-1-phosphate receptor agonist)
Recent pathogenetic concepts Genetics (NOD2, ATG16L1 and many others) Environment (smoking, urban living, antibiotics) Microbiome (“Dysbiosis,” low diversity, pathobionts)

In CD, induction with biologicals ranged from 15% to 40%, and there was also a wide range of therapeutic gain and NNTs (1). At any rate, in both UC and CD, fewer than half of the patients achieved clinical remission, and introducing the new softer endpoint of “response” only improved the graphs and not the situation of the patient, who wants to be well and not just better. Indirect comparisons of the key studies may be compromised by the different patient cohorts recruited, but direct head-to-head studies, such as the Varsity trial, showing the superiority of vedolizumab vs. adalimumab in UC are rare (25). Lacking reliable predictors, it is still up to an individual choice of doctors and patients as to which biological, if necessary, is selected based on the efficacy and side effect spectrum.

Long-term results of both biologicals and the new oral drugs are even worse because there is not only primary nonresponse, but also significant and continuous loss of response. Some of this loss can be attributed to anti-idiotype antibodies, but escape may also be a consequence of other mechanisms. In most controlled trials of biologicals and the new oral medications, nonresponders were usually dismissed and not counted in the final calculation of remission rates (1). Thus, if normalized to the initial population and followed for approximately 1 year, indeed >75% of those initially recruited in the trial do not achieve response or lose their response or remission despite continued treatment. Accordingly, also in “real-life studies” persistence on the biologicals is low (26).

In the seminal top-down trial by D´Haens et al. (27) comparing corticosteroids (low dose of 40 mg/day prednisolone equivalent or budesonide) vs. a combination of infliximab and azathioprine, at the end of the study, remission rates were essentially identical between the groups. As expected, about two thirds of the steroid-treated active-control patients required azathioprine for maintenance, and about 20% required infliximab. By this design, this early study defined the proportions of patients actually in need for a given therapeutic. In concordance with current clinical use, about 20% are refractory to standard treatment and require a biologic or oral new drug. In the REACT study (28) using cluster randomization of gastroenterology practices, the 12-month remission rates were similar at early combined immunosuppression and conventional management practices (66% and 62%). Thus, there is little direct evidence that biologics are superior to the standard corticosteroids/thiopurines in terms of efficacy. However, particularly in refractory patients, in those with extraintestinal manifestations or fistulation in CD and otherwise refractory UC, biologicals make a big difference (1, 24).

Adverse events following anti-TNFs include increased infection rates, particularly activation of latent tuberculosis and other serious complications, such as sepsis, paradoxical psoriasis, probable lymphoma, and rare melanoma (1). Vedolizumab and ustekinumab are better tolerated, whereas the JAK-inhibitors are associated with serious infections, herpes zoster and thrombosis (4). Ozanimod has not increased the infection rate, but increased liver enzymes although the experience is still quite limited (23). Thus, side effects should be considered also in these newer medications but are certainly not prohibitory.

Rather than targeting only patient-reported outcomes, such as activity scores, it has been proposed to “hit hard and early” in order to achieve endoscopic mucosal or even histological healing, and the STRIDE consensus (3) promotes implication of this concept. However, although mucosal healing (MH) is a good prognostic parameter, this does not justify escalating treatment until MH is finally achieved. In addition and unfortunately, MH is only documented in a minority of patients independent of treatments used (1). However, achieving steroid-free “deep remission,” including MH, with infliximab in combination with an immunosuppressant did not prevent progression (29). This seems to apply also to other biologicals because timely escalation of ustekinumab therapy for patients with CD, based on early endoscopic response, clinical symptoms, and biomarkers, did not result in significantly better endoscopic outcomes at week 48 than symptom-driven decisions alone (30). Apparently, the STRIDE approach was a “good old boys (and girls) sitting at a table” consensus lacking evidence at the time, advanced by the industry interested in marketing, and now the trial data actually suggest that there is *no* benefit in forced treatment escalation to achieve MH. This is not surprising because disease progression to fibrosis obviously is more related to microbes than inflammation (31).

Taken together, there is a definite benefit from the use of monoclonal antibodies or the newer oral drugs in those patients who are refractory to the standard therapy or in certain clinical situations (extraintestinal manifestations or fistulae). However, initial lack or later loss of response remain a frequent problem and often require switches until all options have failed with surgery to follow. Therefore, even the most enthusiastic proponents of biologicals accept that there is a therapeutic ceiling that limits treatment success (2). Biologicals and probably also the newer oral drugs have significantly broadened the therapeutic armamentarium in IBD but have failed to achieve permanent remission in most patients and, sadly, also cannot prevent disease progression: there is no cure in sight. Several new JAK-inhibitors and novel antibodies will probably be marketed soon, including anti-IL23, but, as stated above, probably will not revolutionize the field (4, 5). This should motivate us to rethink the therapeutic approach focusing on the novel pathogenetic concepts derived from extensive genetic and microbiological studies and, above all, understanding IBD as a barrier disease (7).

### Recent pathogenetic concepts

#### Genetics

It is estimated that about 12% of patients have a family history of CD, and the overall genetic impact, as calculated from twin studies, is a considerable 20%–50% in CD and 14%–19% in UC (32). Gene-wide association studies (GWAS) have helped in pinpointing more that 240 associated and possibly causal genes or regions (33). It has been possible to fine-map IBD loci to single variant resolution. The analysis of five chronic inflammatory diseases identified considerable overlap and also disease-specific patterns at shared loci (34). Therefore, the genetic background is enormously complex, and many of the genes showing up in the huge statistics exhibit only a very limited impact with a relative of risk of <1.2 (increase of risk by 20% or less): in this case, a positive finding would, for example, only increase the risk from 1 in 500 to 1 in 400.

Even this large and still increasing number of associated genes merely explains about 14% of disease variance in CD and only 7.5% in UC (35), suggesting that there is a considerable and still ill-defined role for epigenetics and other factors (36). About 80%–90% of GWAS-identified loci are confined to noncoding variations and, thus, are only indirectly interfering with gene transcription (35). Further complications arise from the genetic differences with varying ethnic background (37) and, most importantly, different phenotypes of IBD. For example, it was nicely shown that, also genetically, there is a significant difference between ileal and colonic CD (38). Thus, we should categorize three different IBDs and not just two, and this is also reflected by clinical data. It is also suggested that inflammation status modulates the effect of host genetic variation on intestinal gene expression in inflammatory bowel disease so that disease activity remains important as a modifier.

Still, the most important step is from correlation to causation (39), and we, therefore, discuss only a few genes in detail that are a likely cause and also have significant impact on disease risk in CD. Unfortunately, data on likely causal genes are much more limited in UC, in which many are HLA-associated (40, 41), but there is also a significant (67%) overlap with CD risk genes. Also, because of the much smaller impact of genetics in UC, we, therefore, focus on CD. The genetic background in (very) early onset IBD is quite different (40), but the important role of epithelial genetics applies to all age groups (42).

The first causal gene to be identified in CD was nucleotide-binding oligomerization domain-containing protein 2 (NOD2), exhibiting three common variants (fs1007insC, R702W, and G908R) (32, 43). A meta-analysis of 39 studies showed that the odds ratio for simple heterozygotes was 2.4 and for homozygotes/compound heterozygous carriers about 17. NOD2-carriage is specifically associated with ileal involvement, stricturing complications, and a modestly earlier age of onset (32). Each of the polymorphisms leads to a compromised or complete loss of function in this intracellular pattern recognition receptor in binding its ligand bacterial muramyl-dipeptide (MDP). Consequently, the lack of NFκB stimulation upon MDP binding induces, at first sight paradoxically, *hyporesponse* of inflammatory cells, such as macrophages or dendritic cells (39). MDP-binding through NOD2 normally downregulates Toll-like receptor (TLR) expression, a defective signal might, therefore, imply unresponsive TLR-activity. There is also a link of NOD2 with the NLRP3 inflammasome to induce colitis in Nod2 knockout mice. On the other hand, NOD2 mutations limit the production of proinflammatory cytokines; such diminished cytokine production in a CD-associated allele is counterintuitive.

**Figure 4:**
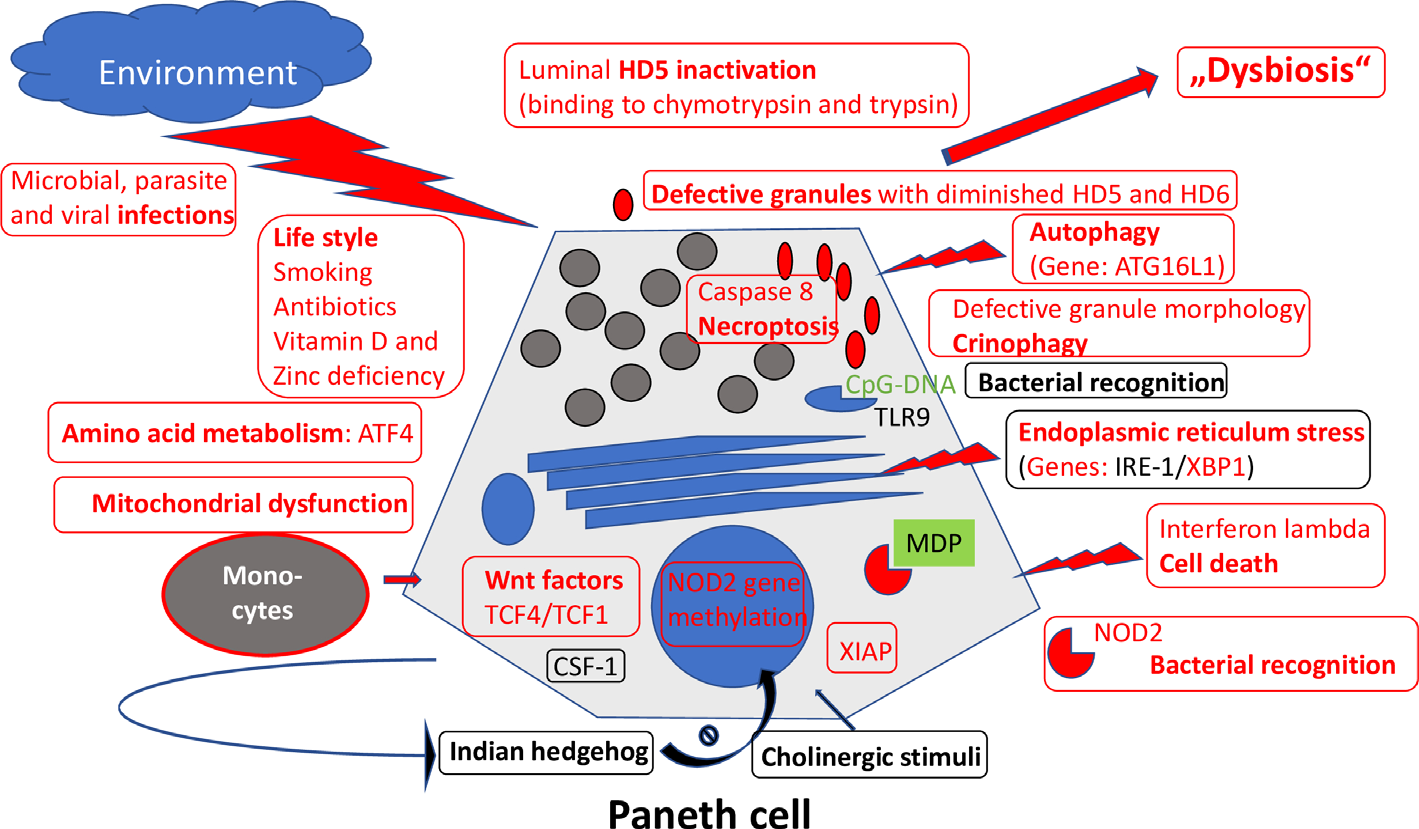
Overview of mechanisms regulating Paneth cell function and morphology. Those defective in ileal CD are labeled in red. Adapted from Wehkamp and Stange (44).

Importantly, the Paneth cells located at the base of the small intestinal crypts and producing antimicrobial defensins to shield against invading bacteria also express significant amounts of NOD2 (**Figure 4**). Most patients with CD of the small intestine but not of the large intestine underproduce α-defensins, and this is particularly pronounced in those with polymorphisms in the NOD2 gene in both European and American patients (45, 46). Among the competing cells potentially responsible for mediating the NOD2 effect, the Paneth cell is the only cell type that explains why these mutations are not associated with CD in general but preferably with ileal disease.

Another CD-associated and probably causal gene is ATG16L1, a component of the autophagy machinery (47, 48). Autophagy is a cellular degradation process for both natural substrates as well as bacteria. Apparently, the disease-associated gene single nucleotide polymorphism (Thr300Ala) forms an unstable and readily degraded protein by introducing a caspase 3 or 7 cleavage sequence (35). The result is an impaired autophagy, likely to play a significant role in CD. Interestingly, NOD2 interacts with ATG16L1 during autophagosome formation. A key cell in which this impaired autophagy may be important is, again, the Paneth cell. Especially by studying the role of endosomal stress and autophagy, it was demonstrated convincingly that Paneth cells are a site of origin of intestinal inflammation (49). In patients with both NOD2 and ATG16L1 CD-associated polymorphisms, the Paneth cell morphology is characteristically impaired (50) and, probably, also its function compromised. Interestingly, in the Japanese population, the ATG16L1 polymorphism is irrelevant, but its role in impairing Paneth cell morphology is taken by LRRK2. Thus, in different ethnic backgrounds, including Asians, the Paneth cell seems to be important at the center of pathogenesis.

In contrast to the abovementioned NOD2 and ATG16L1, which play key functions in innate immunity, polymorphisms of the IL23 receptor complex are indeed protective against both CD and UC. This cytokine plays a central role in regulating the differentiation of CD4+ T cells into proinflammatory Th17 cells (39). These Th17 cells are critical in antimicrobial defenses and also in epithelial functions. However, the realms of adaptive and innate immunity are not completely separate: exposure of γδ-IELs to IL23 promoted IL-22 production, which triggered Paneth cells to secrete angiogenin 4, an antimicrobial peptide (51). Most certainly, any modulation of “adaptive immunity” cytokines, such as IL-22 or IL-23, also impacts innate mechanisms, such as antimicrobial products stemming from Paneth cells. Obviously, the border between adaptive and innate immunity should not be construed as being distinct, and an IL-23-related genetic mechanism is likely to impact also on Paneth cell innate function.

Finally, there is a Mendelian IBD: the X-linked inhibitor of apoptosis (XIAP) deficiency, also known as the X-linked lymphoproliferative syndrome type 2 (XLP-2), a rare primary immunodeficiency. XIAP deficiency is characterized by a key triad of high susceptibility to develop hemophagocytic lymphohistiocytosis frequently triggered by Epstein–Barr virus infection, recurrent splenomegaly, and IBD with the features of Crohn’s disease. Very recently, it was demonstrated that the loss of XIAP rendered Paneth cells sensitive to microbiota-, TNF-, receptor-interacting protein kinase 1 (RIPK1)-, and RIPK3-dependent cell death. This is associated with deficiency in Paneth cell–derived antimicrobial peptides and alterations in the stratification and composition of the microbiota (52).

In conclusion, the complexity of IBD genetics is enormous, and in an overview of pathways, the epithelial barrier, microbicidal mechanisms, and microbe sensing play a dominant role although adaptive immunity is also relevant (40). The key point, however, is that host–microbe interactions have shaped the genetic architecture of IBD (41). It should be noted that despite the enormous progress in IBD genetics, these findings have not translated into improved diagnosis, categorization, or prognosis in clinical practice. To gain further insight into these IBD complexities, the environment is of interest, and in particular, the microbiome as a significant permanent resident within the gut.

#### Environment

There is no doubt that it is the environment that plays the major role in IBD risk, and it, therefore, has received significant attention. In a recent “umbrella review” of meta-analyses (53), nine factors that increase the risk of IBD were identified: smoking (CD), urban living (CD and IBD), appendectomy (CD), tonsillectomy (CD), antibiotic exposure (IBD), oral contraceptive use (IBD), consumption of soft drinks (UC), vitamin D deficiency (IBD), and non-*Helicobacter pylori*-like enterohepatic *Helicobacter* species (IBD). Also, seven factors were identified that reduce risk of IBD: physical activity (CD), breastfeeding (IBD), bed sharing (CD), tea consumption (UC), high levels of folate (IBD), high levels of vitamin D (CD), and *Helicobacter pylori* infection (CD, UC, and IBD). In some instances, reports are conflicting, and ongoing research in the Netherlands (54) has identified four novel factors: stressful life events [CD odds ratio (OR) 2.6/UC OR 2.9], high perceived stress [2.3/2.7], alcohol use [0.4/0.4], and bronchial hyperreactivity [3.0/2.4]. Four novel factors were associated with only CD: prenatal smoke exposure [1.9], having a bed partner [0.53], allergies [2.7], and cow’s milk hypersensitivity [5.9] and two solely with UC: carpet flooring [0.57] and neuroticism [1.3]. Thus, stopping smoking, avoiding antibiotics early in life, having a pet and a bed partner, still avoiding stress, having a drink at night, and carpet flooring are recommended (more serious suggestions to avoid IBD will soon be published by the IOIBD).

These facts probably translate into the rapid increase of IBD as early as the second generation of immigrants from low-incidence countries to a Western country. The critical rise of IBD, both CD and UC, in many developing or Westernizing countries likely also relates to these environmental factors, in particular, the diet (55). Currently, IBD is expected to still rise in Western countries and even more in those adapting their lifestyle accordingly, such as China or Japan. It remains to be seen whether the famous apple a day prevents this dramatic development.

Similar to genetics, as discussed above, it is not trivial to tell association from causation. Smoking´s mechanism of action has long been unclear until it turned out that it interferes with Paneth cell function (56), whereas vitamin D is crucial for epithelial and Paneth cell protection against bacteria (57)): this suggests a direct causal role. Smoke extracts also affect macrophage function directly but actually favor an anti-inflammatory response. Interestingly, the effect of smoking is modified by the genetic background of the patients. On the other hand, early overuse of antibiotics and the diet associated with a Western lifestyle probably are linked to the microbiome and probably act indirectly although this “Western” diet also impairs Paneth cell function (58). A mechanism that is actively involved in mediating environmental influences in IBD is epigenetics (36). For example, butyrate as a bacterial product is involved in epigenetic regulation, but these interesting links are beyond the scope of this review. As an important part of our environment, the microbiome deserves a separate chapter because it is the target of the immune response in IBD rather than an autoimmune response to gut tissue (59).

#### Microbiome

After this fact became apparent, the 1000 species containing a microbiome with four phyla (Bacteriodetes, Firmicutes, Proteobacteria, and Actinobacteria) entered center stage. Particularly, following a metagenomic revolution identifying the “bugs” not through culture (most are not culturable), but through microbial 16S-rRNA analysis and sequencing, the field redirected its focus. At this writing, more than 6490 papers in Pubmed have appeared under the headings “IBD and microbiome.” The key question still is whether the changes found in the microbiome are *primary* or *secondary* in IBD and associated inflammation. For the sake of brevity and extensive previous reviews (60), the discussion is focused on bacteria although there clearly also is a mycobiome and a virome in the gut, and some data are now emerging.

A recent systematic review elaborated that 75% of the CD studies but only 29% in UC showed a significant decrease in the α-diversity in patients with IBD, whereas the rest of the studies found no difference compared with healthy controls (61). Detailed analysis revealed that, among the Firmicutes, 43 distinct ribotypes were identified in the healthy controls compared with only 13 in CD. In this study, the Clostridium leptum group was particularly diminished, whereas the Prevotella subgroup was even increased. Another study from Sweden suggested that the distance to the healthy plane of the fecal microbiome was maximal in ileal CD, somewhat smaller in colonic CD, and minimal in ulcerative colitis (62). In a classic study in new onset Crohn ´s patients, bacterial community membership was associated independently with intestinal inflammation, antibiotic use, and therapy (63). As a caveat, fecal bacteria are affected by such simple variables as stool consistency, suggesting that many of the changes observed may simply be due to diarrhea secondary to IBD. It should also be noted that most of the studies report “relative” data and not the quantitative microbiome profiling that is known to link gut community variation to microbial load. Actually, in both CD and UC, gut microbial density was reduced (64).

In another detailed study, it was noticed that IBD samples were depleted of Lachnospiraceae and Bacteroidetes but enriched for Proteobacteria and Actinobacteria (65). In the profound analysis already quoted above, it was demonstrated that there was significant overlap in a principal component analysis between both IBDs and controls, an axis with an increased abundance of Enterobacteriaceae, Pasteurellacaea, Veillonellaceae, and Fusobacteriaceae and a decreased abundance in Erysipelotrichales, Bacteroidales, and Clostridiales could be defined (63). Notably, this axis correlated strongly with disease status; i.e., inflammation had a significant impact on the microbiota. It was also demonstrated that a superb differentiation could be obtained using the principal bacterial metabolic pathways that were either increased or decreased in CD. Thus, function may well be more important than species composition.

On the other hand, only about a third of Crohn ´s patients harbor adherent-invasive *E. coli* as a “pathobiont” in their mucosa, which survive in macrophages, are clearly pro-inflammatory (66), and are antagonized by Paneth cell HD5. This is contrasted by a protective commensal group, Faecalibacterium prausnitzii, diminished in CD. Again, as mentioned above with regard to diversity, this finding was not completely consistent: 6 of 11 studies in CD and 4 of 10 studies in UC confirmed a significant decrease (61).

This scenario of antagonistic bacteria, of course, is suggestive of a required balance between more aggressive or more defensive (“anti-inflammatory”) species at the mucosal site. Thus, it is rather the ecology that is important and not a single culprit. As discussed, it is also remarkable that gut microbiota may induce not only inflammation, but also fibrosis independent of inflammation (31). This could explain the poor performance of biologicals in preventing progression (see above).

Bacteria are certainly on the crime scene (67), but to come back to the question above (hen or egg), several points have to be considered: the first and probably most important argument against the hen is the important role of inflammation in these microbial changes. In longitudinal studies, dependence on inflammation during a relapse is also obvious (68). In inactive UC, microbial composition was closer to the controls than to active disease (69). Interestingly, colonic microbiota are associated with both inflammation and host epigenomic alterations in IBD (70). There are also reports that, upon long-term remission, the bacterial composition slowly normalizes over time. In a detailed endoscopic study of inflamed (CDI) versus noninflamed (CD-NI) matched biopsy sites (71) CD-I patients showed an altered mucosal microbial community compared with CD-NI patients and controls. Matched biopsy samples in CD-I patients revealed that sites of injury, identified endoscopically, are characterized by increased encroachment of bacteria to host epithelial cells. It should be noted that a microbial difference between inflamed or noninflamed sites or patients is still controversial (61). Second, the reduced diversity discussed above is not specific for IBD and can also be found, for example, in type 2 diabetes. Third, healthy co-twins share the IBD-like microbiome features with their diseased siblings but are not sick (72). Fourth, a favorable therapeutic response, for example, to enteral feeding or anti-TNF, is associated with normalization of the microbial flora (73). Furthermore, experimental inflammation drives dysbiosis in animals. Finally, the gut microbial profile of preclinical CD is similar to that of healthy controls (74). This was independently confirmed by the case of a fecal donor developing CD later, and none of the 31 recipients developed the disease. These, in our opinion are strong arguments against a primary role of the bacterial microbiota.

Also, the individual intestinal bacterial flora (“the enterotype”) is not “self-regulated” and autonomous, but subject to selection by the host and affected by the genetic predisposition (75). Thus, the CD risk genes NOD2 and ATG16L1 as well as endosomal stress genes are known to affect the intestinal microbiome. Avoiding the effect of disease and inflammation on the microbiome, a Dutch group recently demonstrated a significant impact of IBD risk genes on certain bacteria, such as Roseburia, even in healthy individuals (76).

This genetic influence is complemented and maybe sometimes antagonized by the environment and personal characteristics. Interestingly in a large Swiss trial, the major factors in this regard were body mass index and age as well as overall lifestyle, including sport, smoking, and alcohol consumption, which were also correlated with the gut microbiota (77). Probably the key factor accessible to modification is the diet, which is likely impacting on the epidemiology as discussed above. A Westernized diet is the most relevant environmental factor during globalization of IBD, and this is probably mediated at least in part by the microbiome (78). It may be concluded that the intestinal microbiome clearly is the target of intestinal immune response, but there is little evidence for a *primary* role.

The key point of all these immense complexities of genetics, environment, and microbiome (**Table 2**) is the simple fact that none renders a cure perspective. It will be virtually impossible to individually correct these multiple genetic defects, and in UC, the genetic impact is too small to be optimistic even if, with CRISPR/Cas, genetic engineering on this scale might be possible in the future. Similarly, improving the environment to reduce disease risk will also not cure the disease. The most radical way of modifying the intestinal microbiome by fecal transfer has already failed in the majority of UC patients and is rather ineffective in CD (6). This is not unexpected if, as suggested above, the microbiome composition not responsible for disease *initiation*. So what is the alternative central disease mechanism triggering the inflammation that could prove to be the elusive treatment target?

## The future

**Figure 5:**
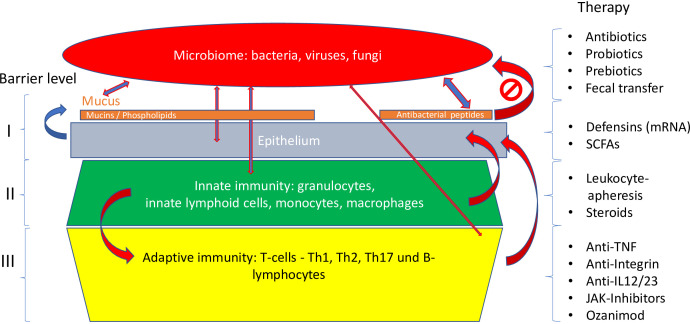
Different levels of the mucosal barrier targeted by respective therapies. Adapted from Stange and Schröder (7).

To answer this central question, a closer look is necessary at the complex barrier (**Table 3**; **Figures 5****–** **7**), at which the confrontation between the mucosal surface and the gut microbes occurs: 10^13^ against 1. The odds are against us, but still, the healthy state is the rule. The first layer of defense is the mucus, which appears to be defective in UC; the second component is the multitude of antibacterial peptides, inadequate in CD. Both are secreted by the epithelium, which, as a physical continuum of cells, is linked by tight junctions. The second layer consists of mucosal innate immune cells, and the third of adaptive immune cells, which are the targets of classical and current therapies. Obviously, these layers represent a secondary response to bacterial invasion as discussed above. Rather, we focus on two key epithelial cells with specialized functions, the goblet and the Paneth cells which offer the opportunity to focus on primary events.

**Table 3:** Key points 3

Novel pathogenetic concepts Defective mucus in UC Incompetent antibacterial barrier in CD
Future therapies and their targets Mucus-enhancing microbes, probiotics Calcium-activated chloride channel regulator 1 Ly6/PLAUR domain containing 8 Goblet cell antibiotic WMCD2 Stimulating mitochondrial function Defensin-fragments mRNA coding for defensins Enhancing goblet and Paneth cell differentiation

### Novel pathogenetic concepts

#### Mucus in health

**Figure 6:**
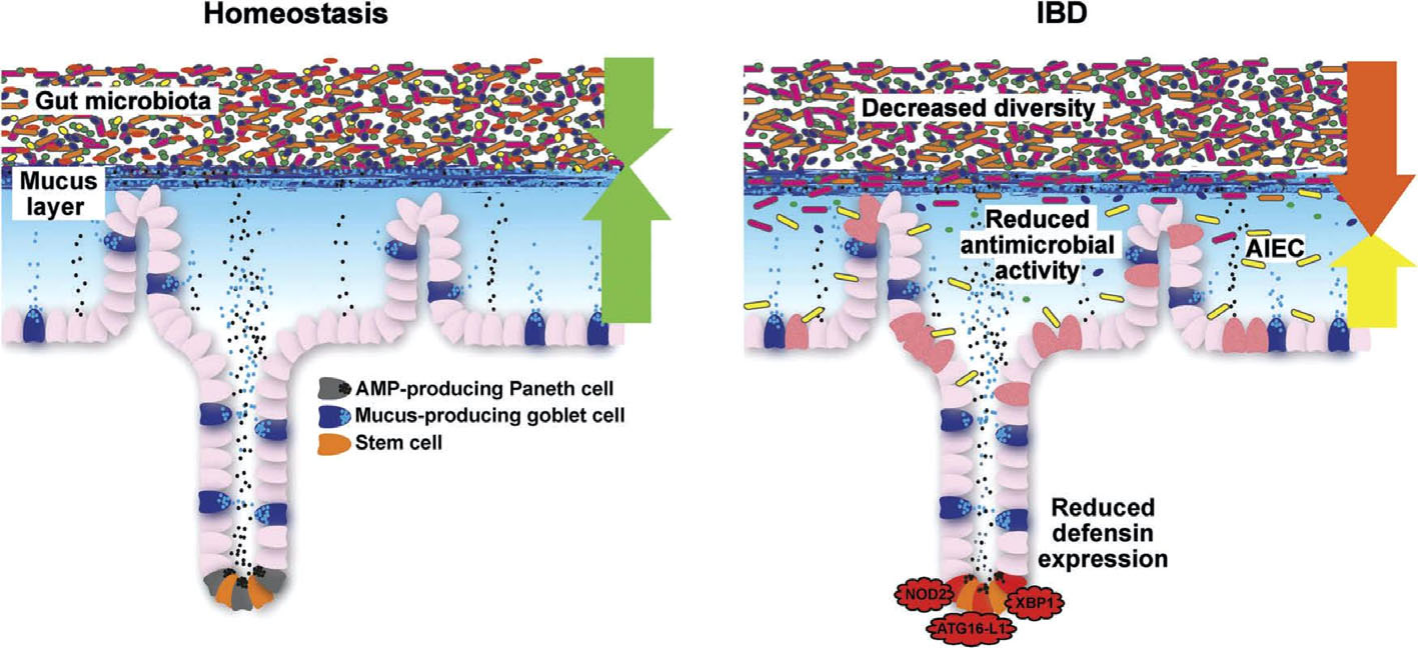
Schematic representation of the normal antibacterial barrier and defective defense in the small intestine. Adapted from Stange and Schröder (7).

**Figure 7:**
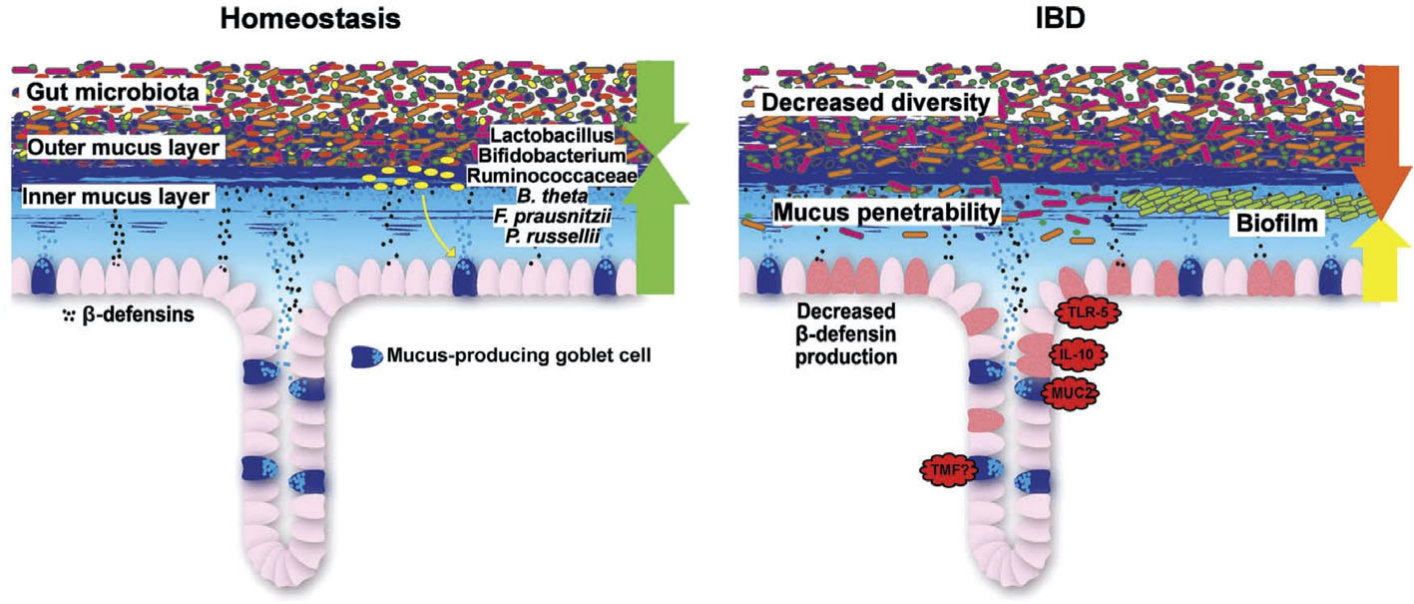
Schematic representation of the normal antibacterial barrier and defective defense in the colon. Adapted from Stange and Schröder (7).

As we detail in a recent review on this topic (7), it is the mucus layer that keeps the commensal microbiome at a distance, protecting the epithelium (**Figures 6**, **7**). Whereas small intestinal mucus is single layered, patchy, and easily removed, the mucus layer in the colon is two-layered, and the inner layer is adherent (79). Quite surprisingly, this inner layer attached to the colonic epithelium is devoid of bacteria in healthy individuals. The mucus structure is made up mostly of MUC2 as well as multiple other components as shown in a detailed proteomic analysis (80). MUC2 is a huge protein (2.7 MDa), which is di- and trimerized from monomers and increases its bulk by swelling after water contact.

Recently, it was revealed that colon mucus consists of two distinct O-glycosylated entities of Muc2: a major form produced by the proximal colon, which encapsulates the fecal material, including the microbiota, and a minor form derived from the distal colon, which adheres to the major form. The microbiota directs its encapsulation by inducing Muc2 production from proximal colon goblet cells. In turn, O-glycans on proximal-derived Muc2 modulate the structure and function of the microbiota as well as the transcriptome of the colon mucosa. These studies demonstrate that the proximal colon, by producing O-glycosylated mucus, acts as a master regulator of host–microbe symbiosis.

Mucus is formed by the goblet cells in both the small and large intestines although their localization is different, being mostly in the crypt or at the surface, respectively (81). There is a sentinel goblet cell at the crypt mouth mediating alarm signals in case of bacterial invasion. Goblet cells receive their signals from various Toll-like receptors (TLR1-5) and, accordingly, are strongly responsive to the bacterial environment. Remarkably, the key cytokine regulator of goblet cells is apparently IL-18, suppressing goblet cell differentiation factors such as Klf4 and SPDEF (82). On the other hand, IL-22 altered the mucus layer *in vitro* by inducing an increase in membrane mucus but a decrease in secreted mucus and goblet cell content. IL-22 had the same effect on antimicrobial peptides and membrane mucus in both healthy and IBD human samples. In contrast, this IL-22-associated epithelial phenotype was different when treatments were performed in the presence of butyrate or immune cells (Goblet cell mechanisms IL-22). Remarkably, goblet cells also actively transfer bacterial antigens to dendritic cells, thus regulating the antibacterial immune response.

Some mucolytic bacteria, such as *Akkermansia muciniphila*, may degrade the protective layer, particularly if the microbiome is deprived of fiber (83). Other microbes such as *Bacteroides thetaiotaomicron*, an acetate producer, have the reverse effect and increase goblet cell differentiation and mucus production (84). The two layers of mucus persist in germ-free animals, but the inner layer becomes penetrable. The so-called Western diet is deleterious to the mucus (85), which probably relates to the epidemiology of UC as mentioned above. Muc2 synthesis is negatively regulated by the Golgi-associated protein TMF/ARA160, and consequently in TMF^-/-^ mice, the colonic mucus is refractory to bacterial penetration (7). Taken together, these and other data imply that, normally, there is a strong interaction, in harmony, of goblet cells and surrounding bacteria. In the normal healthy state, because the inner layer is devoid of bacteria, the microbial influx into the mucosa is likely minimal and easily dealt with by the macrophage and neutrophil system (**Figure 5**): an activation of the basically immune-tolerant adaptive immunity is simply not required.

#### Mucus in UC

Overall, in UC, mucus thickness is indeed reduced (86), whereas in CD, it appears to be normal (86). In those areas with acute inflammation, the mucosa was actually denuded of the mucus layer, suggesting that inflammation further impairs it. A recent study also supports the hypothesis that a weakening of the colonic mucus barrier contributes to the development of UC pathogenesis as the MUC2 protein was significantly reduced independent of local inflammation (87). The concept of a primary role for defective or deficient mucus is supported by the induction of overt colitis in muc2-knockout (MUC2-deficient) mice, indicating that the MUC2-protein is critical for colonic protection.

Low mucus levels may be related to diminished goblet cell numbers in UC but not in CD, whereas mRNA-expression was found to be in a rather normal range, suggesting post-transcriptional regulation (88). Also, structurally, there are UC-related defects of glycosylation and sulfation. In fecal studies, there was at least no obvious difference between controls, CD, and UC with respect to mucus-degrading glycosidase activity although an effect of local and differential bacterial mucolytic activity is possible (83).

A single cell expression analysis comparing epithelial clusters found, even in uninvolved segments, 207 significantly dysregulated genes (89). Therefore, uninvolved mucosa is not simply normal, but “preinflamed.” This may well be linked to the well-known clinical feature of a defined, endoscopically visible border between the inflamed and uninflamed colonic segment, which migrates proximally: possibly, the not yet inflamed mucosa is forced to reduce protective mucus formation by its immediate inflamed neighborhood, including the cytokine milieu (IL18) (82).

These structural defects in UC may directly result in the presence of commensal bacteria in the mucus (90), and some apparently pass the normally impenetrable inner colon mucus layer in both murine colitis models and patients with UC (91). This slow bacterial invasion, in turn, may trigger an *adequate* secondary adaptive immune reaction. Compared with microbiota from healthy donors, microbiota from IBD patients generate biofilms *ex vivo* that are larger in size and cell number, contain higher intracellular iron concentrations, and exhibit heightened virulence in a model of human intestinal epithelia (92).

In addition to being confronted with nearby commensals in UC, the goblet cell is also exposed to a complex and delicate cytokine milieu: during active inflammation, key pro-inflammatory cytokines, such as TNF-α, interferon (IFN)-γ, and IL-13, have direct deleterious effects on epithelial barrier integrity (93). In conclusion, convincing evidence supports the role of mucus in protecting the mucosa during health and its dysfunction or even complete lack as a primary event permitting bacterial invasion. As inflammation in terms of cytokine milieu and bacterial influx is deleterious to goblet cells, this is likely a vicious cycle with tissue destruction as a result.

It remains to be elucidated why goblet cells are diminished and do not function adequately, but some concepts have evolved. Some years ago, we suggested that the differentiation factors driving the intestinal stem cell to differentiate into a goblet cell are normally induced by inflammation, and this was functioning in CD (94). However, in UC, none of these factors was upregulated, which would explain both low goblet cell numbers as well as compromised function. It seems quite possible that this is related to the IL-18 hyperactivity mentioned above.

In the single cell study quoted above (89), there were important additional findings, including major changes in the goblet cell clusters and, importantly, a reduced expression of the crypt-based goblet cell–associated peptide WFDC2. The peptide is bactericidal and downregulated in UC, and a heterozygous knockout results in bacterial penetration and adhesion to the epithelium. Goblet cells also produce other antibacterials, such as Reg1a, but overall, the antimicrobial peptide (AMP) response in UC is characterized by an induction of epithelial defensin and cathelicidin formation (95); more details are given below. Such an induction of defense may actually be counterproductive by eliminating beneficial bacteria as was shown experimentally by knocking out RELMß and, indirectly, the antimicrobial lectin RegIIIß (96). Consequently, the goblet cell–derived mediator RELM-β then drives spontaneous colitis in Muc2-deficient mice, possibly by promoting commensal microbial dysbiosis.

The old Roediger paradigm of UC as an energy-deficiency disease of the colonic epithelium has been revived (93). Looking at over- vs. under-expressed genes in UC, Haberman et al. (97) counted 3600 and 1696, respectively, and many of the latter were mitochondrial. In UC, there is a loss of mitochondrial homeostasis (including mitophagy and the autophagic removal of damaged mitochondria), which can lead to defective energy production and increased mitochondrial oxidative stress (93). In a recent study in UC mucosa, the mitochondrial p32 was related to oxidative phosphorylation in upper colonic crypts and suppressed in UC inflammation (98). In contrast to the crypts, which rely on glycolysis, the upper crypts and surface survive by ß-oxidation of bacteria-derived short chain fatty acids. The low p32 not only compromised energy supply, but was also related to goblet cell differentiation. Thus, the mitochondrial defects relate to the UC defect in the stem-to-goblet cell transition discussed above.

Mitochondrial DNA is not only known for its role in cellular energetics and oxidative phosphorylation, but is also an agonistic player in the innate immune system (93). Upon degradation of the mitochondrial membrane, this rather stable and through epigenetic changes “foreign looking” DNA interacts with TLRs and NODs, resulting in cytokine stimulation. Apparently, mitochondrial DNA is a proinflammatory damage-associated molecular pattern released during active IBD.

#### Mucosal antibacterial peptides in health

In functional tests, the normal colonic mucus layer exhibits a strong antimicrobial activity (99). Accordingly, we detected antimicrobial peptides in mucus extracts from healthy persons, including the defensins HBD-1 (human ß-defensin 1), HBD-2, HBD-3, and the cathelicidin LL-37, ubiquitin, lysozyme, histones, high mobility group nucleosome-binding domain-containing protein 2, olfactomedin, ubiquicidin, and other ribosomal proteins. AMPs are bound electrostatically by mucins, but inhibition of antibacterial activity was limited, and therefore, binding must be reversible. The abovementioned fact that the inner mucus layer is virtually devoid of bacteria is not only due to the physical barrier of mucins, but also of the AMPs reversibly bound to the mucus.

AMPs are ubiquitous but organ-specific: as mentioned above, in the small intestine, Paneth cells mediate genetic and environmental impact in IBD (**Figure 4**). They act as “maestros of the crypt (100) by supporting and, if necessary, redifferentiating into the neighboring stem cells. They clear the crypt and mucosal surface from bacteria by production of multiple AMPs, including the α-defensins HD5 and HD6 as well as lysozyme, angiogenin 4, and various lectins, including the REGs (44). The new kid on this block is intelectin 2 (101). All these peptides exhibit different bacterial specificities. Most AMPs act directly on the bacterial cell wall in their natural form or after physiological reduction of disulfide bridges by thioreductase. Some, such as HD6 and HBD1, can, in addition, form nanonets to block the crypt lumen and prevent bacterial attack on the stem cell. Defensins, such as HD5 and HD6, not only act as intact peptides, but may, under physiological conditions, disintegrate and explode like a “bombshell” into multiple shorter peptides with differential antibacterial activities toward several different strains. In addition to being antibacterials, most AMPs also exhibit immune-modulatory or chemoattractant functions, increase epithelial proliferation, or induce mucus formation.

The presence of functional Paneth cells is essential in resistance against several enteric pathogens, including Salmonella and Shigella, whereas Clostridium difficile infections, frequent in IBD, inhibit Paneth cell function as a means to persist in the gut (102). Although there is some controversy, probably the key secretagogues for the Paneth cell are IL-17A, IL-22 from intraepithelial lymphocytes with downstream IL-18, and interferon-γ in addition to bacterial components (103). Sometimes, degranulation seems to be coupled to complete cell extrusion and Paneth cell loss although the long Paneth cell life span of about 30 days suggests that this happens infrequently.

In the colon, important AMPs (95, 99, 104) are the constitutive HBD1, the inducible HBD2 and 3 as ß-defensins, cathelicidins (in humans LL37), the lectin REGIII (regenerating islet-derived protein 3), and RELM (resistin-like molecules). Elafin (or skin-derived anti-leukoprotease) and secretory leukocyte protease inhibitor (SLPI) are additional “defensin-like” molecules with broad antimicrobial activity. These peptides act in concert to protect the mucosa from bacterial invasion of commensals as well as pathobionts, and many are induced during infections or inflammation. There is also evidence that they govern the composition of the luminal microbiome in humans (105) although the experimental animal studies are much more extensive to prove this point in both the small and large intestines. In the colon, epithelial cells are responsible for AMP-synthesis but, in some instances, also neutrophils and other inflammatory cells. Although there are a few Paneth cells also in the healthy cecal area and there is also a “Paneth-like” cell in the normal colon, their function is unclear and probably not comparable to the small intestine.

#### Mucosal antibacterial peptides in CD

In this chapter, we focus on CD, because, in UC, the AMP system appears to be functioning (with the possible exception of WFDC2). The relevance of the AMP system is apparent because it is tightly linked to the disease localization in the individual CD patient, a feature unlike any other pathogenetic hypothesis. Therefore, we discuss ileal or ileocecal and colonic CD separately. The comprehensive updated evidence covering the role of the Paneth cell in ileal CD was recently published with the provocative title “Paneth´s disease” because ileal disease is increasingly recognized as a separate entity (38, 44).

In the beginning, there was the surprising observation that NOD2 was intensely expressed by the Paneth cell (**Figure 4**). As noted above, in two cohorts in Germany and the United States, the Paneth cell α-defensins HD5 and HD6 mRNA and protein were diminished in small intestinal but not colonic CD (45, 46). The low HD5 was not related to inflammation, but to the NOD2 genetic status. Similarly, low HD5 expression levels were also observed in younger (<18 years) patients (44). More recently, in an unsupervised expression analysis of American pediatric patients, again, HD5 expression was reduced related to enhanced interferon-γ and tended to be particularly low in NOD2-mutated children at age >10 years (106). Intelectin 2 was also found to be low in ileal CD (independent of genetics) (101). In Australian patients, low HD5 in ileal CD was confirmed but related to inflammation and not NOD2.

In contrast, other Paneth cell peptides, such as lysozyme or sPLA2 (secretory phospholipase A2) were not diminished in the small intestine (45, 46), implying that the Paneth cell number was probably not impaired. Histological studies have demonstrated that, even in noninflamed CD segments, there is a redistribution of Paneth cells to the crypt mouth but low counts in the lower crypt (107). Finally, the Wnt system as a key regulator of Paneth cell differentiation was less active in CD (44), suggesting that, in IBD, the stem cell differentiation to Paneth cells is restricted.

These findings are complemented by morphological observations of deranged Paneth cell granules and crinophagy in ileal CD (50, 108). These aberrations likely reflect functional impairment and, as detailed above, were related to the established genetic links of ileal CD (50). As mentioned above, there is additional genetic evidence implicating the Paneth cell: mutations or deficits in NOD2 and the autophagy gene ATG16L1 as well as genes of endosomal stress. Environmental influence from a Western diet as well as smoking negatively impact the Paneth cell and may induce ileal inflammation as well as microbiome changes. Paneth cell alertness to pathogens is maintained by vitamin D receptors, and vitamin D levels are frequently low in CD. A recent paper implied that mitochondrial impairment drives intestinal stem cell transition into dysfunctional Paneth cells predicting CD recurrence (107), a nice analogy to energy problems of goblet cells. Thus, functional, genetic, morphological, and environmental data support the conclusion that ileal CD is associated and probably caused by a specific cell resulting in “Paneth´s disease” (44, **Figure 4**).

There is some evidence that colonic CD also may be associated with a defective defensin system as suggested many years ago (95) although the data are not as comprehensive as in ileal CD. Colonic CD is characterized by low HBD1, regulated by PPARγ, and a compromised induction of HBD2, HBD3, and cathelicidine (109). This is associated with a low mucosal antibacterial activity against B. vulgatus and E. faecalis but not S. aureus. Notably, another interesting peptide called bactericidal/permeability increasing protein (BPI) is demonstrated to be linked to the disease course in UC: lower levels are associated with a more severe disease course. Independent studies confirmed this defective induction in CD compared with UC. It is unclear whether this defect is related to the absence of inductor stimuli such as butyrate, Muc2, or even vitamin D. Possibly as an additional defense, metaplastic Paneth cells may appear in the inflamed IBD colon although it is also conceivable that they increase damage-deleting beneficial bacteria. It may be concluded that the role of Paneth cell defensins and Paneth cell alterations are well established in ileal CD, whereas the colonic AMPs and their defects require more research.

## The barrier as treatment target

### Targeting mucus

The different types of therapy available or being developed address various levels of mucosal protection and immunity (**Figure 5**). Earlier attempts to target mucus by enhancing mucus formation and epithelial energy by supplying additional short chain fatty acids locally were inconclusive (110). Another approach was the oral administration of lecithin to combine with the mucus, but it failed in phase III (111).

An attractive concept is the use of mucus-enhancing microbes, such as *Lactobacillus rhamnosus* or *reuteri* (7), to stabilize this protective layer by enhancing mucus production and restoring goblet cell numbers. *Bacteroides thetaiotaomicron* is known to increase goblet cell differentiation and may also be therapeutic. Another candidate in this regard is *Peptostreptococcus russellii*, which colonizes the mucus and also increases goblet cell numbers (7). It may also be possible to achieve enhanced goblet cell differentiation; candidates in this regard are prebiotics or synbiotics, and until now, results of clinical trials have been mixed. Probiotics, such as *E. coli* Nissle, of course, have been used successfully in UC before but were limited to maintaining remission.

Other potential targets include calcium-activated chloride channel regulator 1 (CLCA1), a metalloprotease, and TMF/ARA169. CLCA1 is known to contribute as an endogenous mucosal factor to the mucus growth rate and, if activated, could minimize bacterial penetration (7). As discussed above, TMF/ARA160 is a negative regulator of Muc2 synthesis, and its blockade by an siRNA-inhibitor could stabilize mucus and also promote its formation (112).

Lacking a proper mucus structure to retain AMPs, probably their stimulation will not succeed in UC therapy (with the possible exception of *E. coli Nissle*). The same limitation may apply to Ly6/PLAUR domain containing 8 (Lypd8), which is reported to promote the segregation of flagellated microbiota as well as pathogenic bacteria from colonic epithelia (113). Substituting for the missing goblet cell antibiotic WMCD2 should have the same limitations unless parallel production of mucus by these cells is achieved. Finally, it has been successfully proven that the mitochondrial and energy approach may be used by giving a specific diet, at least in animals (98). This may be unpalatable to humans, but changing the diet rather than giving a chronic medication is attractive. In conclusion, there are several strategies to follow, and with some optimism, targeting the mucus in UC is quite promising.

### Targeting antimicrobial peptides

The easiest way to substitute defensins or other AMPs is to administer them orally, and this has been done successfully in experimental animals (114). Alternatively, defensin-inspired peptidomimetics or defensin fragments could be used. However, considering the evidence arguing against a chronic antibiotic treatment in IBD, there is little optimism in acting from the lumen. A much smarter avenue would be to deliver mRNA as capsules to the epithelial cells normally producing these peptides. Wrapped in ileal or colonic release galenics, which are available, it may be possible to stimulate local synthesis.

The alternative option would be to enhance AMP formation by administration of the natural triggers for synthesis, such as interleukins, such as IL-22, whereas interferon-γ would probably be counterproductive by initiating cell extrusion. Similarly, blocking interferon λ with inhibitors of the JAK-Stat pathway, such as filgotinib (in development for CD), was shown to block Paneth cell death (115). Also, some probiotics are reported to induce ß-defensins, but there is little evidence in CD for a beneficial effect. Enhancing Paneth cell differentiation looks like a promising option, but stimulating Wnt unspecifically may be associated with tumor promotion elsewhere.

A revolutionary approach has been taken in Japan (116), where it has proven possible to transplant living intestinal organoids into the gut. If this could be reproduced in humans, the local defense by AMP synthesis could be strengthened. There will be major obstacles, including graft versus host, and most likely, repeated administrations will be necessary, but it appears to be technically feasible.

Finally, also in the Paneth cell, the mitochondria link was used to stimulate cell function (107). Reinforcing mitochondrial respiration by inhibition of glycolysis restored inflammation-imprinted dysfunction of the stem cell niche. In another study with a similar focus on mitochondria, the addition of mito-tempo, a mitochondria direct antioxidant was shown to normalize innate function of Paneth cells (117). These findings clearly demonstrate that the mitochondrial damage is reversible and offer a new chance to intervene. Bile acids are shown to be toxic to Paneth cells and, accordingly, sequestrants have proven useful in protecting their function (118). Finally, the supply of sufficient vitamin D and adhering to a reasonable (non-Western)! diet as well as stopping smoking will probably be helpful, the latter at least in CD. It remains to be seen whether any of these promising approaches will be successful.

## Conclusion

Looking back, the approach to IBD has largely been empiric and preferred the techniques that were new and exciting at the respective times. Histology led to cell identification, discovery of cytokines revolutionized the inflammation field, and finally genome and bacterial metagenome analysis opened new frontiers. When chemistry allowed the construction of new molecules, sulfasalazine was designed and worked, devoid of any logic. The introduction of corticosteroids and immunosuppressants was based on the vague concept of an immune-mediated disease but also meant trial and error with many failures, such as ciclosporine or mycophenolate in CD. In the next step, the construction of monoclonal antibodies introduced new “biological” therapies, and there was no dearth of targets: some, such as etanercept, had the right target but failed anyway, and others, such as anti-IL17, unpredictably made things worse. Finally, recognition of the key roles of the microbiome and the various barrier defects in IBD will lead to new treatment candidates, but still, successful drug development remains tedious and open-ended. And we should not forget that the aim is curing IBD and not improving a questionable primary outcome (119) or revenues.

## Author contributions

The author confirms being the sole contributor of this work and has approved it for publication.

## Acknowledgments

This overview is based on many years of intense scientific cooperation and discussions with many leaders in the IBD-field, both national and international. Initial eye openers to the defensin area as well as close partners were the pioneers J.M. Schröder, Kiel and C. Bevins, Davis. The contribution of many coworkers and friends in the laboratory was outstanding and special thanks go to J. Wehkamp, K. Fellermann, K. Herrlinger, B. Schröder, M. Gersemann and S. Nuding. The generous funding by the Robert Bosch Foundation and, much later, also the Deutsche Forschungsgemeinschaft is gratefully acknowledged. This article is dedicated to D.E. Stange and M. A. Klose.

## Conflict of interest

ES has received honoraria for consulting Amgen, CureVac, Dr. Falk Pharma, Janssen, Merck und Takeda and has given lectures supported by AbbVie, Dr. Falk Pharma, Ferring, Janssen and Takeda.

## Publisher’s note

All claims expressed in this article are solely those of the authors and do not necessarily represent those of their affiliated organizations, or those of the publisher, the editors and the reviewers. Any product that may be evaluated in this article, or claim that may be made by its manufacturer, is not guaranteed or endorsed by the publisher.
